# Assessment of bone mineral density and bone metabolism in young men with obstructive sleep apnea: a cross-sectional study

**DOI:** 10.1186/s12891-022-05644-8

**Published:** 2022-07-16

**Authors:** Jian-Ming Zhao, Bi-Ying Wang, Jie-Feng Huang, Han-Sheng Xie, Meng-Lan Chen, Gong-Ping Chen

**Affiliations:** 1 Department of Respiratory and Critical Care Medicine, Fujian Province, the First Affiliated Hospital of Fujian Medical University, NO 20, Chazhong road, Taijiang district, Fuzhou, 350005 People’s Republic of China; 2 Fujian Provincial Sleep-Disordered Breathing Clinic Center, Fujian, China; 3 Institute of Respiratory Disease, Fujian Medical University, Fujian, China

**Keywords:** Obstructive sleep apnea, Bone mineral density, Bone metabolism, Young men

## Abstract

**Background:**

Hypoxia in obstructive sleep apnea (OSA) patients during sleep may have an effect on bone metabolism. Few data regarding evaluation of bone metabolism in young individuals diagnosed with OSA. In this study, we aim to identify the association between bone mineral density and OSA in young men (≤ 40 years old of age).

**Methods:**

Consecutive male subjects who underwent polysomnography were enrolled. Serum calcium, 25-hydroxyvitamin-D3, β-isomerized form C-terminal telopeptide of type I collagen, osteocalcin and procollagen type 1 N-propeptide were measured in all participants, and bone mineral density (BMD) at lumbar spine (L1-L4), femoral neck and hip total were determined by dual energy X-ray absorption (DXA).

**Results:**

The population consisted of 85 subjects (mean age 35.53 years). The BMD at lumbar spine (L1-L4) in moderate OSA patients was higher than control and severe OSA group significantly (*p* = 0.036). After adjustment for confounding factors, stepwise multiple linear regression analyses showed LaSO_2_ (β = 0.340, *p* = 0.008) as an independent explanatory variable for Lumbar L1–L4 BMD, LaSO_2_ (β = 0.304, *p* = 0.037), BMI (β = 0.393, *p* = 0.008) for femur neck BMD and BMI (β = 0.720, *p* = 0.002) for hip total BMD. Conclusions.

Our finding indicated that there was a relationship between OSA and bone metabolism in younger men, and moderate OSA-related hypoxia positively related with BMD. This study also showed that different degrees of recurrent hypoxia had different effects on bone metabolism, a finding that required further investigation.

## Background

Obstructive sleep apnea (OSA) is a common sleep disorder characterized by sleep disruption, recurrent apnea, and intermittent hypoxia during sleep, affecting approximately 4% of men and 2% of women in the general population [1]. It results in oxidative stress, endothelial dysfunction, inflammation, and enhanced sympathetic activity, which may lead to many pathological consequences, including cardiovascular, endocrine, and respiratory and neurocognitive dysfunction [2, 3]. Additionally, intermittent hypoxia may play an important role in determining the consequences of OSA.

Hypoxia is also known to downregulate osteogenic differentiation and directly stimulate osteoclast formation and bone resorption [4, 5]. A recent meta-analysis indicated that OSA is strongly associated with an increased risk of osteoporosis in cohort studies [6]. Animal studies have suggested that 5 h of chronic intermittent hypoxia stimulates early mobilization of mesenchymal stem cells (MSC) from the bone marrow into the blood circulation [7], allowing their differentiation into various types of cells such as osteoblasts and endothelial progenitor cells [8, 9]. Therefore, intermittent hypoxemia during sleep in patients with OSA may be a risk factor for osteoporosis, which is a skeletal disorder that may become a serious health problem characterized by pain, loss of independence, a reduced quality of life, and a high risk of fractures [10]. However, Mariani and colleagues [11] found no correlation between apnea–hypopnea index (AHI) and bone mineral density (BMD) in moderate and severe obese OSA groups. Similarly, the study of Sforza et al. [12] found decreased BMD in elderly patients but not in middle-aged patients. However, the small number and non-homogeneous age range of the patients were weak points in these studies. Thus, whether OSA affects bone metabolism, especially in young men, remains unclear.

The aim of our study was to examine whether or not OSA is associated with changes in BMD after adjustment for relevant covariates such as age, sex, weight, and metabolic and inflammatory indices in a cohort of young male patients.

## Methods

### Subjects

Consecutive young men (≤ 40 years of age) who presented to our sleep unit between January 2016 and January 2017 for assessment of sleep disturbances, such as habitual snoring, witnessed sleep apnea, and excessive daytime sleepiness state were recruited. All participants provided written consent prior to the study, and the study was approved by the Ethics Committee of the First Affiliated Hospital of Fujian Medical University. All subjects were asked to completed a questionnaire containing history of smoking and alcohol consumption, illness, and medical treatment. Current smoker was defined those who had a smoking history and had not quit smoking. Daytime sleepiness was assessed by using a Chinese version of the Epworth Sleepiness Scale (ESS) [13]. Patients previously diagnosed with or treated for OSA were excluded. We also excluded patients reporting a history of a disease inducing pathological fractures, taking any drugs known to affect bone metabolism or the endocrine system, hyperparathyroidism, inflammatory disorders and any chronic condition that could influence bone metabolism.

### Anthropometric and biochemical measurements

Weight and height were measured to the nearest kilogram and centimeter, respectively, with the patients wearing light clothing and in bare feet. The body mass index (BMI) was defined as body weight in kilogram (kg) divided by height*height in square meter(m^2^). Neck circumference was measured by a measure tape in the middle of the neck between the mid-cervical spine and mid-anterior neck, and waist circumference was measured in the middle between the lowest rib and top of the iliac crest at the end of gentle expiration. Blood pressure was gauged using a standard mercury sphygmomanometer on the right arm in a sitting position with a 1-min interval after a 5-min rest. The average of two readings was used for the analyses.

### Polysomnography

All the subjects underwent nocturnal polysomnography (P Series Sleep System; Compumedics; Melbourne, Australia) from 22:00 to 06:00 to obtain the recording of electrooculography(EOG), electroencephalography(EEG), submental electromyography(EMG), and electrocardiography. Beside, thermistors for nasal and oral airflow, thoracic and abdominal, impedance belts for respiratory effort, pulse oximetry for oxyhemoglobin saturation at the finger tip were also recorded. All polysomnography findings were scored by a physician on the basis of the criteria in the 2012 American Academia of Sleep Medicine (AASM) manual [14]. By definition, obstructive apnea was defined as the absence of airflow for at least 90% from baseline for at least 10 s. Obstructive hypopnea was defined as a 30% or greater reduction in airflow lasting 10 s or longer, and combined with a 4% or more decrease in oxyhemoglobin saturation. The apnea–hypopnea index (AHI) was defined as the average number of apneic plus.

hypopneic episodes per hour of sleep. Apnea and hypopnea time index (AHTI) was calculated as the percentage of total sleep time spent with apnea and hypopnea. The oxygen desaturation index (ODI) was defined as the total number of episodes of oxygen saturation (SpO_2_) ≥ 4% per hour of electroencephalographic sleep time. We also recorded lowest O_2_ saturation (LaSO_2_) and the percentage of total sleep time spent with SpO_2_ less than 90% (T90%). According to AHI, OSA severity was classified into three groups: an AHI of < 10 events/h was classified as the control group, an AHI of 10 to < 50 events/h was classified as the moderate OSA group, and an AHI of ≥ 50 events/h was classified as the severe OSA group [15].

### Biochemical measurements

Fasting blood samples were obtained from all subjects in the morning after the PSG recording to assess the serum concentrations of glucose, lipid profile including total cholesterol (TC), low density lipoprotein-cholesterol (LDL-C) high density lipoprotein-cholesterol (HDL-C) and triglycerides (TG), creatinine, calcium and phosphorus levels. All parameters were measured with the H-7600 autoanalyzer (Hitachi, Tokyo, Japan). A BNII nephelometer (Dade Behring, Deerfield, IL, USA) was uses to detect high-sensitivity C-reactive protein (Hs-crp) was measured usin. The electrochemiluminescence method was applied to measure plasma concentrations of osteocalcin, β-isomerized form carboxy-terminal telopeptide of type I collagen (β-CTx), 25-hydroxyvitamin-D3 (25-OH-D), and procollagen type I N-propeptide (PINP) through the Cobas® kit (Roche, Germany) in the Cobas e601® analyzer (Roche, Germany) [16].

### BMD measurement

The BMD of every participant at the lumbar spine (L1–L4), the femoral neck and hip total was evaluated by using a dual-energy X-ray absorptiometry (DEXA) (Hologic ASY-00409) scan. And BMD was expressed as g/cm^2^ and t score.

### Statistical analysis

The study data were analyzed using SPSS 19.0 (SPSS Inc, Chicago, IL, USA). All p values were two-sided, and all results were considered statistically significant at p < 0.05.

All descriptive data were tested for normal distribution before statistical analysis. The data were expressed as mean ± standard deviation (SD), median (interquartile range) and proportion (percentage) in the cases of normally distributed, skewed and categorical data, respectively. For normally distributed continuous variables, one-way analysis of variance, followed by post hoc comparisons was used to compare the data between the three groups. For data that were not distributed normally, Kruskal–Wallis H (K) was performed for multiple-group comparison. Categorical data were analyzed by Chi-square test or Fisher’s exact test. Spearman’s coefficient was performed to examine linear correlation between variables. Multiple linear regression analysis was used to determine the independent predictors of BMD. A stepwise variable selection method was performed in the multivariate analysis.

## Results

Overall, 145 young men were enrolled in this retrospective study. Forty-six were excluded because they met the exclusion criteria, and 14 were excluded because they did not provide consent. Finally, 85 young men were included. The mean age and BMI was 35.53 ± 4.53 years (ranged from 24 to 40 years), 27.75 ± 3.81 kg/m2 (range, 20.98–41.65 kg/m^2^), respectively. Based on the AHI, OSA (AHI ≥ 10 events/h) were found in 70 (82.35%) patients. All patients were then further subdivided into a control group (AHI of 0 to < 10 events/h, *n* = 15), moderate OSA group (AHI of 10 to < 50 events/h, *n* = 27), and severe OSA group (AHI of ≥ 50 events/h, *n* = 43).

The anthropometric and polysomnographic characteristics of the patients were summarized in Table 1. There were no significant differences among the three groups in terms of age, antidiabetic agents and current smoking. Polysomnographical parameters including AHI, ODI, T90%, AHTI and ESS score increased significantly with OSA severity, whereas LaSO_2_ decreased significantly with an increase in OSA severity (all *p* < 0.05). In addition, severe OSA patients seem to be heavier when compared with control group (29.30 ± 3.92 kg/m^2^ vs. 23.69 ± 2.17 kg/m^2^, *p* = 0.000).

**Table 1:** Comparison of clinical characteristics and polysomnographic parameters according to OSA severity

	Controls	Moderate OSA	Severe OSA	*p* values
Subjects (n)	15	27	43	
Age	33.60 ± 5.12	34.96 ± 4.16	36.45 ± 4.30	0.082
Antihypertensive agents, number (%)	1/15(6.7)	4/27(14.8)	17/43(39.5)	0.012*
Antidiabetic agents, number (%)	0/15(0.0)	2/27(7.4)	4/43(9.3)	0.403
Current smoking, number (%)	3/15(20.0)	12/27(44.4)	15/43(34.9)	0.364
Neck circumference (cm)	38.61 ± 2.62	40.50 ± 3.06	42.33 ± 5.07	0.012*
Waist circumference (cm)	90.79 ± 9.46	95.11 ± 7.13	100.29 ± 8.84	0.001*
BMI (kg/m^2^)	25.39 ± 3.13	26.92 ± 2.25	29.25 ± 3.74	0.000*
AHI	6.80(5.30,9.20)	34.15(20.10,43.68)	69.62(58.10,80.35)	0.000*
ODI	3.40(1.30,8.15)	23.85(14.45,30.31)	64.15(49.03,71.20)	0.000*
T90%(%)	0.06(0.00,0.42)	2.50(0.25,5.86)	20.38(8.83,33.04)	0.000*
LaSO_2_ (%)	88.00(84.00,90.25)	80.50(69.25,87.00)	61.50(49.75,72.50)	0.000*
AHTI	2.19(1.19,3.49)	14.66(10.03,23.73)	31.12 (27.28,40.65)	0.000*
ESS score	5.00(3.00,12.00)	6.50(5.00,10.00)	10.00(6.00,14.50)	0.024*

*OSA* Obstructive sleep apnea, *BMI* Body mass index, *AHI* Apnea–hypopnea index, *ODI* Oxygen desaturation index, *T90%* the percentage of total sleep time spent with SpO2 < 90%, *LaSO*_*2*_ lowest O_2_ saturation, *AHTI* Apnea and hypopnea time index, *ESS* score Epworth Sleepiness Scale score, *p was considered statistically significant

The laboratory parameters were shown in Table 2. The three groups did not differ significantly in TC, TG, HDL-C, LDL-C, fasting glucose, creatinine, glycated hemoglobin.

**Table 2:** Laboratory parameters of patients with OSA and control subjects

	Controls	Moderate OSAS	Severe OSAS	*p* values
Subjects (n)	15	27	43	
TC (mmol/L)	4.66 ± 0.62	5.09 ± 0.79	5.01 ± 0.82	0.505
TG (mmol/L)	1.04(0.57,2.44)	1.81(1.22,3.73)	1.97(1.45,3.06)	0.497
HDL-C (mmol/L)	1.12 ± 0.22	1.01 ± 0.16	0.97 ± 0.23	0.418
LDL-C (mmol/L)	3.19 ± 0.26	3.57 ± 0.82	3.38 ± 0.77	0.600
Fasting glucose (mmol/L)	4.57 (4.37–4.91)	5.02(4.72–5.71)	5.20 (4.80–5.74)	0.054
Creatinine(μmol/L)	64.00 ± 17.69	72.11 ± 14.41	73.65 ± 13.29	0.327
glycated hemoglobin	5.20 (3.77–5.65)	5.30(5.10–6.10)	5.50(5.30–5.83)	0.224
Hs-crp(mmol/L)	0.57(0.30,6.86)	1.06(0.61,1.86)	2.53(1.07,6.64)	0.008*

Normally distributed data were expressed as mean ± SD, skewed data (including TG, HDL-C and fasting glucose) were presented as median (interquartile range)

*OSA* Obstructive sleep apnea, *TC* Total cholesterol, *TG* Triglycerides, *HDL-C* High-density lipoprotein-cholesterol, *LDL-C* Low-density lipoprotein-cholesterol, *Hs-crp* High-sensitivity C-reactive protein, *p was considered statistically significant

The high-sensitivity C-reactive protein level was significantly higher in patients with OSA than in the control group (*p* = 0.008).

Table 3 shown that there were significant differences in Lumbar L1–L4 BMD among the three groups (*p* = 0.036). However, no difference was observed between severe OSA group and control group. Conversely, there were no significant differences in calcium, phosphorus, 25-OH-D, β-CTx, osteocalcin, PINP, femoral neck BMD, total hip BMD, lumbar t score, femoral neck t score, or total hip t score among the groups.

**Table 3:** Comparison of bone metabolism parameters of patients with OSA and control subjects

	Controls	Moderate OSA	Severe OSA	*p* values
Subjects (n)	15	27	43	
Calcium (mmol/L)	2.33 ± 0.16	2.27 ± 0.09	2.24 ± 0.10	0.506
Phosphorus (mmol/L)	1.18 ± 0.04	1.15 ± 0.13	1.11 ± 0.24	0.879
25-(OH) vitamin D (ng/ml)	20.37 ± 7.78	20.34 ± 6.93	19.66 ± 6.61	0.931
β-CTx (ng/ml)	0.59 ± 0.15	0.51 ± 0.16	0.46 ± 0.18	0.243
Osteocalcin (ng/ml)	21.34 ± 7.29	21.47 ± 4.67	19.23 ± 5.80	0.337
PINP (ng/ml)	47.39 ± 15.82	53.18 ± 16.49	42.74 ± 16.59	0.096
Lumbar L1–L4 BMD (g/cm2)	1.10 ± 0.14	1.28 ± 0.24	1.14 ± 0.17	0.036*
Femur neck BMD (g/cm2)	0.96 ± 0.11	1.02 ± 0.10	1.00 ± 0.12	0.463
Hip total BMD (g/cm2)	0.97 ± 0.11	1.02 ± 0.09	1.06 ± 0.12	0.360
Lumbar L1–L4 t score	0.37 ± 1.07	0.27 ± 0.97	-0.19 ± 1.41	0.379
Femur neck t score	-0.09 ± 1.04	-0.08 ± 0.61	-0.15 ± 1.04	0.968
Hip total t score	-0.17 ± 0.87	0.19 ± 0.69	0.58 ± 0.95	0.355

*OSA* Obstructive sleep apnea, *β-CTx* β-isomerized form C-terminal telopeptideof type I collagen, *PINP* Procollagen type I N-propeptide, *BMD* Bone mineral density,  *p was considered statistically significant

The correlations between BMD in the lumbar region (L1–L4), femoral neck, and total hip and the anthropometric, polysomnographic, and biological parameters were shown in Table 4. There were significant and positive correlations between lumbar L1–L4 BMD and LaSO_2_ (*r* = 0.291, *p* = 0.023). BMD at the femoral neck and hip total were significantly correlated with BMI and waist circumference (both *p* < 0.05). In addition, BMD at hip total significantly associated with AHI (*p* < 0.05).

**Table 4:** Correlation of sleep disorder parameters and laboratory parameters with BMD at the lumbar spine (L1–L4), the femoral neck and hip total

	Lumbar L1–L4 BMD r(p)	Femur neck BMD r(p)	Hip total BMD r(p)
Age	-0.043(0.714)	-0.128(0.349)	0.025(0.909)
Neck circumference	-0.077(0.546)	0.260(0.053)	0.274(0.196)
Waist circumference	-0.050(0.698)	0.312(0.019*)	0.415(0.044*)
BMI	0.031(0.810)	0.319(0.017*)	0.457(0.025*)
AHI	-0.205(0.107)	0.110(0.419)	0.413(0.045*)
ODI	-0.205(0.115)	0.036(0.799)	0.346(0.098)
T90%	-0.192(0.142)	0.039(0.780)	0.269(0.204)
LaSO_2_	0.291(0.023*)	0.102(0.462)	-0.161(0.451)
AHTI	0.002(0.987)	0.056(0.706)	0.181(0.459)
ESS score	-0.138(0.281)	0.054(0.691)	0.376(0.070)
TC (mmol/L)	0.165(0.284)	-0.152(0.350)	-0.063(0.861)
TG (mmol/L)	-0.021(0.891)	0.191(0.237)	0.486(0.057)
HDL-C (mmol/L)	-0.238(0.140)	-0.206(0.227)	-0.038(0.888)
LDL-C (mmol/L)	0.206(0.201)	-0.253(0.137)	-0.235(0.380)
Fasting glucose (mmol/L)	0.128(0.407)	-0.207(0.201)	-0.393(0.132)
Creatinine(μmol/L)	0.079(0.609)	0.114(0.484)	0.295(0.268)
Glycated hemoglobin	0.038(0.804)	-0.100(0.532)	-0.240(0.309)
Hs-crp(mmol/L)	-096(0.491)	-0.002(0.992)	0.309(0.152)

*OSA* Obstructive sleep apnea, *BMD* Bone mineral density, *BMI* Body mass index, *AHI* Apnea–hypopnea index, *ODI* Oxygen desaturation index, *T90%* the percentage of total sleep time spent with SpO2 < 90%, *LaSO*_*2*_ lowest O_2_ saturation, *AHTI* Apnea and hypopnea time index, *ESS* Score Epworth Sleepiness Scale score TC total cholesterol, *TG* Triglycerides, *HDL-C* High-density lipoprotein-cholesterol, *LDL-C* Low-density lipoprotein-cholesterol, *Hs-crp* High-sensitivity C-reactive protein, *p was considered statistically significant

Stepwise multiple linear regression analyses were performed to explore predictors of BMD at the lumbar L1–L4, the femoral neck and hip total. These analyses showed that LaSO_2_ (β = 0.340, *p* = 0.008) was an independent explanatory variable for Lumbar L1–L4 BMD, that LaSO_2_ (β = 0.304, *p* = 0.037) and BMI (β = 0.393, *p* = 0.008) were independent explanatory variables for femoral neck BMD, and that BMI (β = 0.720, *p* = 0.002) was an independent explanatory variable for total hip BMD (Table 5).

**Table 5:** Stepwise multiple regression analysis of all patients using BMD at the lumbar spine (L1–L4), the femoral neck and hip total as dependent variables

Dependent variable	Independent variable	β	*p* values
Lumbar L1–L4 BMD	LaSO_2_	0.340	0.008*
(R^2^ = 0.116, *P* = 008*)			
Femur neck BMD	LaSO_2_	0.304	0.037*
(R^2^ = 0.156, *P* = 016*)	BMI	0.393	0.008*
Hip total BMD	BMI	0.720	0.002*
(R^2^ = 0.519, *P* = 002*)			

*OSA* Obstructive sleep apnea, *BMD* Bone mineral density, *LaSO*_*2*_ Lowest O_2_ saturation, *BMI* Body mass index,  *p was considered statistically significant

## Discussion

Although there have been several studies showing the effect of OSA on bone metabolism and risk of osteoporosis, few studies have characterized the relationship between the severity of OSA and bone metabolism in young men. The present study was designed to evaluate the association between OSA and BMD in young men as well as the effect of recurrent apnea-related hypoxemia on bone metabolism as evidenced by the change of BMD via DEXA. We found that after adjustment for confounding factors, LaSO_2_ was the independent predicator of BMD at the lumbar L1–L4 and the femoral neck but not of BMD at hip total. Another interesting finding of our study is that the BMD in moderate OSA group was higher than that in the control and severe OSA group, indicating that moderate intermittent hypoxia might play a protective role in terms of bone metabolism to some extent.

OSA is a systemic inflammatory disease that causes abnormalities in cardiopulmonary function and is involved in oxidative stress, endothelial dysfunction, increased sympathetic activity, and metabolic abnormalities [17–19]. Additionally, the mechanisms responsible for hypoxia, increased oxidative stress, and inflammation may affect bone metabolism in patients with OSA [20]. To the best of our knowledge, few studies have focused on the relationship between OSA and bone metabolism. Tomiyama et al. [20] found that severe OSA was associated with increased bone resorption independent of BMI according to the serum/urinary levels of bone resorption markers, and that this association was reversed by continuous positive airway pressure (CPAP) treatment. Additionally, Uzkeser et al. [21] revealed an increased risk of osteoporosis in 21 patients with OSA compared with control patients. Moreover, a population-based cohort study in Taiwan showed that patients with OSA had a 2.74 times higher risk of osteoporosis than patients without OSA [22]. The study by Vilovic M et al. [23] shown that the level of inactive matrix Gla protein were significantly higher in patients with OSA and correlate with disease severity, which indicating that OSA had effect on bone metabolism. However, another study involving a large sample of elderly subjects showed that apnea-related intermittent hypoxia may exert a protective role with regard to age-related decline in bone density by stimulating the bone remodeling process in older population [12]. Therefore, we examined the relationship between the severity of OSA and bone metabolism in young men. Our data showed that young men with severe OSA had lower BMD than young men with moderate OSA, suggesting that more severe hypoxia affects bone metabolism. Many studies have shown that hypoxia affected bone cell function, including increased synthesis of vascular endothelial growth factor (VEGF), insulin-like growth factor II and transforming growth factor b1, decreased expression of the osteogenic transcription factor Runx2/Cbfa1, and increased release of ATP, which are frequently associated with osteopenia [24–27]. However, we also found that the BMD in the moderate OSA group was higher than in the control group, implying that OSA-related hypoxia may be a protective factor for BMD to some extent. A study of Fukuoka et al. [27] showed that when cultured in 10% O2 for 6 days, the numbers of osteoclasts were about 1.6-fold higher than those cultured in 20% oxygen, and the peak of osteoclast formation ranged from 5 to 10% oxygen; osteoclast formation was completely suppressed at 0% oxygen. Notably, one early study examining the influence of oxygen tension on bone resorption parameters found that a moderate reduction in the partial pressure of oxygen (10%) was associated with decreased release of 3H-proline from cultured calvaria, whereas severe hypoxia (30–50%) had the opposite effect [28]. Therefore, it seems reasonable that oxygen tension acts as a significant regulator of bone resorption, and the results of our study seem to support this hypothesis. Further studies are needed to determine the exact role of intermittent hypoxia in bone metabolism.

In our study, we also found that BMI was a significant factor affecting BMD at the femoral neck and hip total. Several studies on the relationship between obesity and bone metabolism have been conducted, and the results were conflicting. Obesity may reportedly reduce BMD through mechanical load-bearing and production of cytokines and hormones [29, 30]. Sforza et al. [12] showed that daily energy expenditure (DEE), ODI and BMI were the most significant factors affecting BMD and the risk of osteoporosis. However, Mariani and his co-workers [11]found that no significant differences between AHI and BMD in obese patients. Our study found that BMI independently affected the femoral neck and total hip BMD which may because the hip and femur are the weight-bearing joints and the BMI is a more important factor on BMD. Factors such as sample size, population, and study design may be responsible for the discrepancies in study outcomes, and further large, prospective cohorts of obese patients with OSA are needed to understand the true effect of obesity and BMI on bone structure.

This study has several limitations that require further evaluation. First, our study was a cross-sectional study and thus could not directly clarified the causal relationship between OSA and BMD. A long-term follow-up study is needed to assess the effect of intermittent hypoxia on bone metabolism. Second, the sample size of the present study was relatively small, and our result was limited to male patients aged < 40 years; thus, extrapolation to women and other age groups is not possible. Third, the patients we enrolled had severe OSA and a relatively high BMI, which might confuse the relationship between OSA and BMD. However, after adjustment for BMI, OSA was still significantly associated with BMD in the lumbar region (L1–L4). Lastly, we did not know the physical activity habits of all subjects, which may some effects on BMD.

## Conclusions

In conclusion, our study showed a relationship between OSA and bone metabolism in younger men, and moderate OSA-related hypoxia positively related with BMD. For a better understanding of this complex relationship, further studies are needed to clarify the effect of the degree of recurrent hypoxia on bone metabolism.

## Data Availability

The datasets used and analysed during the current study are available from the corresponding author on reasonable request.

## Abbreviation

OSA: Obstructive sleep apnea
MSC: mesenchymal stem cells
AHI: hypopnea index
AHTI: Apnea and hypopnea time index
BMD: bone mineral density
BMI: body mass index
ODI: oxygen desaturation index
SpO2: oxygen saturation
LaSO2: lowest O2 saturation
TC: total cholesterol
TG: triglycerides
HDL-C: high density lipoprotein-cholesterol
LDL-C: ow density lipoprotein-cholesterol
Hs-crp: High-sensitivity C-reactive protein
25-OH-D: Plasma 25-hydroxyvitamin-D3
β-CTx: β-isomerized form C-terminal telopeptide of type I collagen
PINP: procollagen type I N-propeptide
VEGF: vascular endothelial growth factor
DEE: daily energy expenditure
